# A visualized dynamic prediction model for overall survival in patients diagnosed with brain metastases from lung squamous cell carcinoma

**DOI:** 10.1111/crj.13625

**Published:** 2023-04-29

**Authors:** Min Liang, Mafeng Chen, Shantanu Singh, Shivank Singh, Caijian Zhou

**Affiliations:** ^1^ Department of Respiratory and Critical Care Medicine Maoming People's Hospital Maoming China; ^2^ Department of Otolaryngology Maoming People's Hospital Maoming China; ^3^ Division of Pulmonary, Critical Care and Sleep Medicine Marshall University Huntington West Virginia USA; ^4^ City Hospital Shahjahanpur India; ^5^ Department of Respiratory Medicine Xinyi Second People's Hospital Maoming China

**Keywords:** brain metastasis, lung squamous cell carcinoma, nomogram, prognosis, SEER, survival

## Abstract

**Introduction:**

Patients presenting with brain metastases (BMs) from lung squamous cell carcinoma (LUSC) often encounter an extremely poor prognosis. A well‐developed prognostic model would assist physicians in patient counseling and therapeutic decision‐making.

**Methods:**

Patients with LUSC who were diagnosed with BMs between 2000 and 2018 were reviewed in the Surveillance, Epidemiology, and End Results (SEER) database. Using the multivariate Cox regression approach, significant prognostic factors were identified and integrated. Bootstrap resampling was used to internally validate the model. An evaluation of the performance of the model was conducted by analyzing the area under the curve (AUC) and calibration curve.

**Results:**

A total of 1812 eligible patients' clinical data was retrieved from the database. Patients' overall survival (OS) was significantly prognosticated by five clinical parameters. The nomogram achieved satisfactory discrimination capacity, with 3‐, 6‐, and 9‐month AUC values of 0.803, 0.779, and 0.760 in the training cohort and 0.796, 0.769, and 0.743 in the validation cohort. As measured by survival rate probabilities, the calibration curve agreed well with actual observations. There was also a substantial difference in survival curves between the different prognostic groups stratified by prognostic scores. For ease of access, the model was deployed on a web‐based server.

**Conclusions:**

In this study, a nomogram and a web‐based predictor were developed to assist physicians with personalized clinical decisions and treatment of patients who presented with BMs from LUSC.

## Graphical abstract

A prognostic nomogram for lung squamous cell carcinoma patients having brain metastases. The nomogram shows the 3‐, 6‐, and 9‐month probabilities of overall survival in this population, which could assist physicians with personalized clinical decisions and treatment of patients.

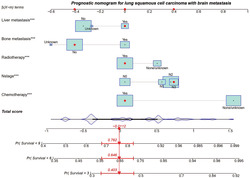

**Abbreviations**
AJCC: American Joint Committee on Cancer
AUC: area under the curve
BM: brain metastasis
DCA: decision curve analysis
LUSC: lung squamous cell carcinoma
NCI: National Cancer Institute
OS: overall survival
ROC: receiver operating characteristic
SEER: Surveillance, Epidemiology, and End Results

## 1 INTRODUCTION

Globally, lung cancer continues to be an enormous challenge to public health. According to the report of the Global Burden of Disease Study, over 2.26 million new cases and 2.04 million deaths were attributable to lung cancer in 2019.^1^, ^2^ As one of the most common histologic subtypes of non‐small cell lung cancer (NSCLC), the overall prevalence of lung squamous cell carcinoma (LUSC) is estimated to be 30%–40%.^3^ In comparison to other sub‐types of NSCLC, LUSC has a limited targeted treatment option and a poor clinical outcome.^4^, ^5^ The majority are diagnosed with metastatic disease or advanced cancer, generally due to a lack of typical clinical symptoms.^6^

Brain metastasis (BM) is a grave complication and a serious challenge to therapeutic strategies for advanced‐stage LUSC. Studies show that about 25%–40% of patients have developed a BM throughout their disease course.^7^ Patients with positive epidermal growth factor receptor (EGFR) gene mutation are particularly susceptible to BMs, with BM prevalence ranging from 44% to 63%.^8^ Despite major developments in cancer treatment over the last few decades, the prognosis for patients who were diagnosed with BMs remains grim.^9^ Most patients had a very poor outcome, with less than 1‐year overall survival (OS) due to chemoresistance,^10^ placing a significant burden on their family caregivers, nursing staff, and society in general.

Nowadays, the TNM staging system of the American Joint Committee on Cancer (AJCC) is one of the most widely applied systems for cancer staging and is an essential tool for guiding treatment and determining prognosis for cancer patients. However, the TNM staging system may be insufficient for predicting LUSC prognosis, since patients with a similar TNM stage and histological classification often have different outcomes.^11^ Furthermore, other prognostic variables than those that define the TNM stages are not incorporated into the system, so factors that might contribute to more accurate prognostication are thus neglected. Thus, sole reliance on the traditional staging system is insufficient and unable to meet patient needs. The devastating prognosis in patients who developed BMs further underscores the importance of an accurate staging system that can precisely predict prognosis and help implement appropriate treatment. In recent years, prognostic nomograms have been regarded as reliable methods for quantifying risks for cancer survival, which can assist clinicians in developing effective treatment strategies and improving patient outcomes. Compared with the TNM staging system, nomograms outperformed in deriving more precise risk predictions and model visualization. To date, no prognostic nomogram has been particularly developed for patients who were diagnosed with BMs from LUSC.

Therefore, in this study, with the data extracted from the Surveillance, Epidemiology, and End Results (SEER) database, we attempted to develop a nomogram to assess the survival probability at 3‐, 6‐, and 9‐month intervals in this population. Furthermore, we compared our nomogram with the TNM staging system developed in parallel to verify the model's performance. Finally, a visualized web‐based nomogram was developed to facilitate its usability.

## 2 METHODS

### 2.1 Compliance with ethics guidelines

This article was built on open‐access databases and did not contain any new research involving human participants or animals. The ethics committee approved the protocol for the Maoming People's Hospital study. All authors have signed the SEER Research data agreement to protect the privacy of patients, which is consistent with ethical principles.

### 2.2 Patient and data selection

The data source of this retrospective cohort study was based on the SEER database (SEER, https://seer.cancer.gov). The SEER program is a national‐based database established by the National Cancer Institute (NCI), which consists of 18 population‐based cancer registries in the United States. Therefore, there is a good representation of clinicopathology, tumor features, and therapeutic information.

The inclusion criteria of the present study were (i) patients with pathologically confirmed LUSC between 2000 and 2018, (ii) patients diagnosed only with primary tumors without multiple primary tumors elsewhere, and (iii) patients with BMs confirmed at initial diagnosis. Exclusion criteria were as follows: incomplete demographic information such as age, sex, ethnicity, and marital status; incomplete clinicopathologic information such as the histologic type, tumor size (defined as the most accurate measurement of a solid primary tumor in the millimeter), primary tumor site, tumor laterality, degree of tumor differentiation, bone, liver, or lung metastasis, and the TNM stage; incomplete therapeutic information regarding chemotherapy and radiotherapy; missing information regarding survival status and follow‐up.

All patient data in this study was extracted from the SEER database with SEER*Stata Software (version 8.4.0.1; https://seer.cancer.gov/data-software/).

### 2.3 Statistical analysis

The primary endpoint was the OS, which was defined as the time interval between the date of the cancer diagnosis and the date of death from any cause. For clinical and demographic characteristics presented at baseline, in the case of continuous variables, the mean and standard deviation were calculated, whereas in the case of categorical variables, frequencies and percentages were calculated. To develop and validate the model, all the eligible patients were randomly divided into two individual cohorts for training and validation at a ratio of 7:3 through computer‐generated random numbers. The training cohort data was applied to develop a nomogram and a classification system for risk assessment. In contrast, the data obtained from the validation cohort was adopted to verify the performance of the model.

Prognostic factors influencing survival were identified using the Cox proportional hazard model. Specifically, univariate Cox regression analysis was performed to select parameters associated with OS. Significant parameters identified in univariate Cox regression analysis (*P* < 0.05) were incorporated into multivariate Cox regression analysis to determine independent prognostic factors and explore their effects. After that, a novel nomogram was developed to predict the 3‐, 6‐, and 9‐month OS among the patients based on these independent factors.

The receiver operating characteristic (ROC) curve, calibration curve, and decision curve analysis (DCA) were conducted to evaluate the performance of the model. Model discrimination was assessed by the area under the curve (AUC) of the ROC curve. A greater AUC value is regarded as a more accurate prognostication. To test the internal validity of this model, we employed 1000 bootstrap resamples to obtain overfitting (optimism) bias‐corrected estimates of prediction performance. Calibration refers to the agreement between predicted probabilities and true probabilities, indicating the extent to which expected and observed outcomes agree. In a perfectly calibrated model, the predictions shall fall on or around the diagonal 45° line of the calibration plot. Finally, DCAs were conducted to evaluate the clinical utility of this model by calculating the net benefits based on threshold probabilities.

A risk classification system was developed according to the aggregate scores of each patient with LUSC in the training cohort by applying the nomogram to separate patients into two prognostic groups, the low‐ and high‐risk groups. Kaplan–Meier (KM) curves were plotted using the median risk score in the data set and a cutoff value to compare the survival risks of high‐risk groups and low‐risk groups.

All tests were performed using the R software (version 4.0.2, https://www.r-project.org/). Statistical analysis was conducted using a two‐tailed test, with *P* < 0.05 considered statistically significant. The following R packages were applied in the model development: “regplot,” “survivalROC,” “caret,” “ggdca,” “foreign,” and “rms.” The “DynNom” R package was used for web‐based dynamic nomogram development.

## 3 RESULTS

### 3.1 Patient characteristics

Of 52 065 patients with LUSC assessed for eligibility, 1812 patients met our inclusion criteria and were enrolled in the study. Of the enrolled patients, 1272 and 540 patients were randomly allocated to the training and validation cohorts for model construction and validation, respectively. At a median survival time of 4 months, 1773 deaths (97.8%) were observed in the total cohort. White people (80.96%), males (63.91%), and the elderly (75.22%) constituted the majority of the cohort. The location of the primary tumor site exceeding a frequency of 50% occurred in the upper lobes of the lungs and was followed by a location in the lower lobes. Over 50% of the tumors ranged from 3.1 to 7 cm in size, whereas over 27% were more than 7 cm and 17% were less than 3 cm. Distant sites of metastasis were as follows: liver metastasis (16.94%), bone metastasis (25.82%), and lung metastasis (22.57%). Over 70% of patients received radiotherapy, and the proportion of patients treated with chemotherapy was slightly higher than that of those who did not receive it. The characteristics between the training and validation cohorts were comparable in terms of baseline demographics, disease characteristics, and treatment history (Table 1).

**TABLE 1:** Demographics, clinicopathologic characteristics, and treatment information of the enrolled patients.

Characteristics	Level	Training cohort	Validation cohort	*P* value
		(*n* = 1272)	(*n* = 540)	
Age, *n* (%)				0.093
	≤40 years	11 (0.865)	0 (0.000)	
	41–50 years	63 (4.953)	18 (3.333)	
	51–60 years	255 (20.047)	102 (18.889)	
	61–70 years	473 (37.186)	196 (36.296)	
	71–80 years	361 (28.381)	175 (32.407)	
	≥81 years	109 (8.569)	49 (9.074)	
Sex, *n* (%)				0.330
	Male	822 (64.623)	336 (62.222)	
	Female	450 (35.377)	204 (37.778)	
Race, *n* (%)				0.355
	White	1022 (80.346)	445 (82.407)	
	Black	181 (14.230)	74 (13.704)	
	Others	69 (5.425)	21 (3.889)	
Marriage, *n* (%)				0.332
	Married	662 (52.044)	285 (52.778)	
	Unmarried	570 (44.811)	231 (42.778)	
	Unknown	40 (3.145)	24 (4.444)	
Histologic type, *n* (%)				0.324
	Papillary Squamous cell	2 (0.157)	0 (0.000)	
	Squamous cell (general)	1191 (93.632)	498 (92.222)	
	Keratinizing squamous cell	42 (3.302)	23 (4.259)	
	Nonkeratinizing squamous cell	30 (2.358)	12 (2.222)	
	Basaloid squamous cell	7 (0.550)	7 (1.296)	
Primary tumor site, *n* (%)				0.252
	Main bronchus	66 (5.189)	35 (6.481)	
	Upper lobe	758 (59.591)	300 (55.556)	
	Middle lobe	49 (3.852)	31 (5.741)	
	Lower lobe	384 (30.189)	167 (30.926)	
	Overlapped lobes	15 (1.179)	7 (1.296)	
Tumor laterality, *n* (%)				0.458
	Left	554 (43.553)	225 (41.667)	
	Right	718 (56.447)	315 (58.333)	
Tumor size, *n* (%)				0.890
	≤3 cm	222 (17.453)	95 (17.593)	
	3.1–5 cm	369 (29.009)	147 (27.222)	
	5.1–7 cm	329 (25.865)	143 (26.481)	
	≥7.1 cm	352 (27.673)	155 (28.704)	
Grade, *n* (%)				0.163
	Grade I	10 (0.786)	3 (0.556)	
	Grade II	216 (16.981)	83 (15.370)	
	Grade III	457 (35.928)	223 (41.296)	
	Grade IV	17 (1.336)	3 (0.556)	
	Unknown	572 (44.969)	228 (42.222)	
T stage, *n* (%)				0.308
	T1	116 (9.119)	46 (8.519)	
	T2	397 (31.211)	189 (35.000)	
	T3	346 (27.201)	150 (27.778)	
	T4	413 (32.469)	155 (28.704)	
N stage, *n* (%)				0.076
	N0	285 (22.406)	142 (26.296)	
	N1	127 (9.984)	59 (10.926)	
	N2	652 (51.258)	241 (44.630)	
	N3	208 (16.352)	98 (18.148)	
Bone metastasis, *n* (%)				0.063
	Yes	319 (25.079)	149 (27.593)	
	No	933 (73.349)	389 (72.037)	
	Unknown	20 (1.572)	2 (0.370)	
Liver metastasis, *n* (%)				0.436
	Yes	214 (16.824)	93 (17.222)	
	No	1033 (81.211)	441 (81.667)	
	Unknown	25 (1.965)	6 (1.111)	
Lung metastasis, *n* (%)				0.692
	Yes	289 (22.720)	120 (22.222)	
	No	957 (75.236)	412 (76.296)	
	Unknown	26 (2.044)	8 (1.481)	
Radiotherapy, *n* (%)				0.056
	Yes	1019 (80.110)	411 (76.111)	
	No/unknown	253 (19.890)	129 (23.889)	
Chemotherapy, *n* (%)				0.964
	Yes	684 (53.774)	291 (53.889)	
	No/unknown	588 (46.226)	249 (46.111)	
Status, *n* (%)				0.232
	Alive	24 (1.887)	15 (2.778)	
	Death	1248 (98.113)	525 (97.222)	
Survival time, median (IQR)	NA	4.000 (2.000, 9.000)	4.000 (2.000, 9.000)	0.851

Abbreviations: IQR, inter‐quartile range; NA, not applicable.

### 3.2 Univariate and multivariate analyses

**FIGURE 1:**
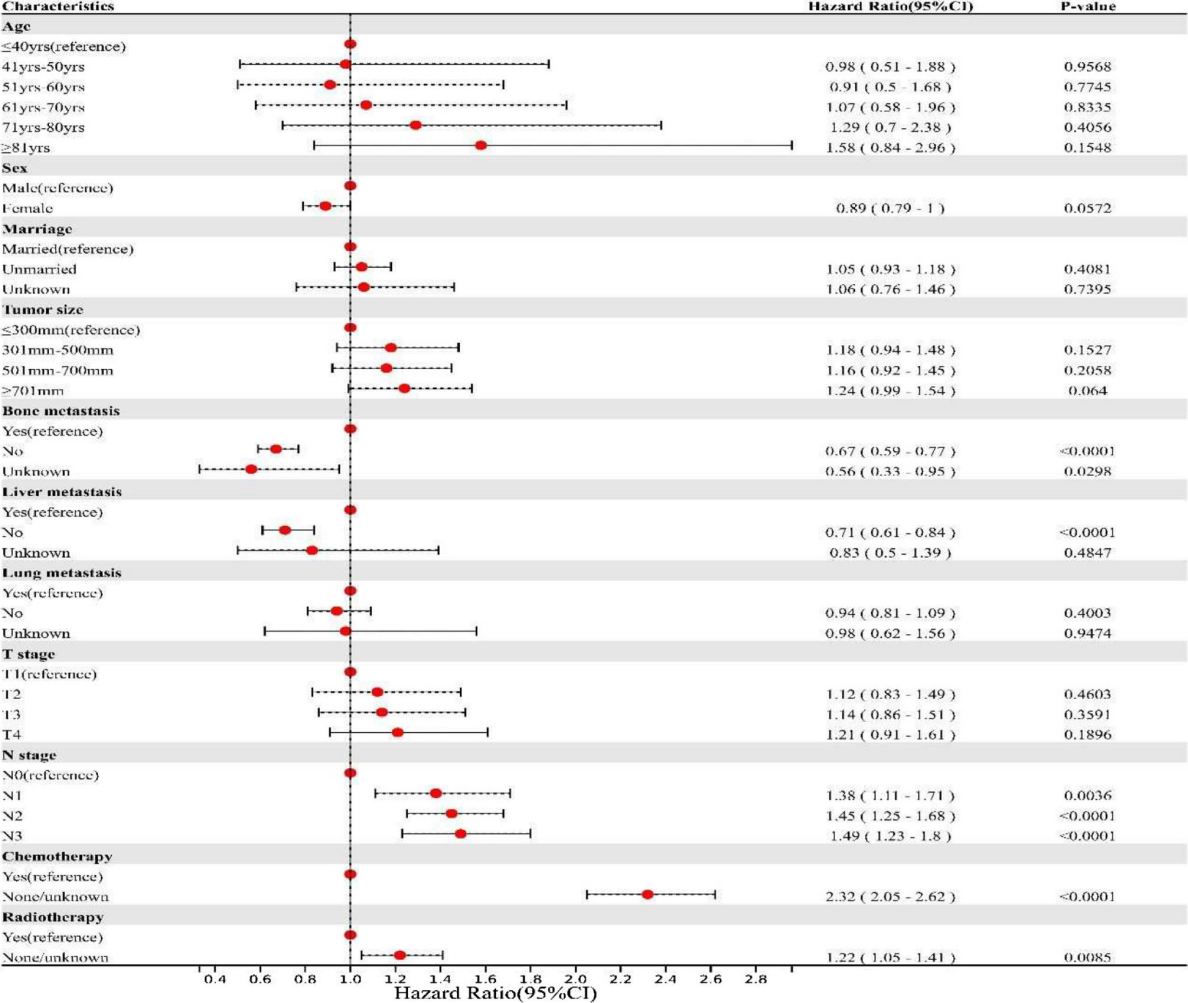
Multivariate COX proportional hazards regression analysis for evaluating prognostic factors for patients who presented with brain metastases from lung squamous cell carcinoma.

We included parameters such as age, sex, marriage, ethnicity, histologic type of tumors, primary tumor site, tumor size, tumor laterality, tumor cell differentiation, distant metastases (bone, liver, and lung), T stage, N stage, chemotherapy, and radiotherapy as covariates in the univariate regression analysis. The significant factors obtained by univariate Cox proportional hazards analysis (*P* < 0.05) were further introduced into the Cox proportional hazards model for multivariate analysis. Results showed that tumor cell differentiation, histologic type of tumors, and primary tumor site were rejected by the univariate Cox proportional hazards analysis. Multivariate Cox proportional hazards analysis further revealed that N stage, bone metastasis, liver metastasis, radiotherapy, and chemotherapy were associated with patients' OS. The results of the multivariate Cox proportional hazards analysis are detailed in Figure 1.

### 3.3 Prognostic nomogram development

**FIGURE 2:**
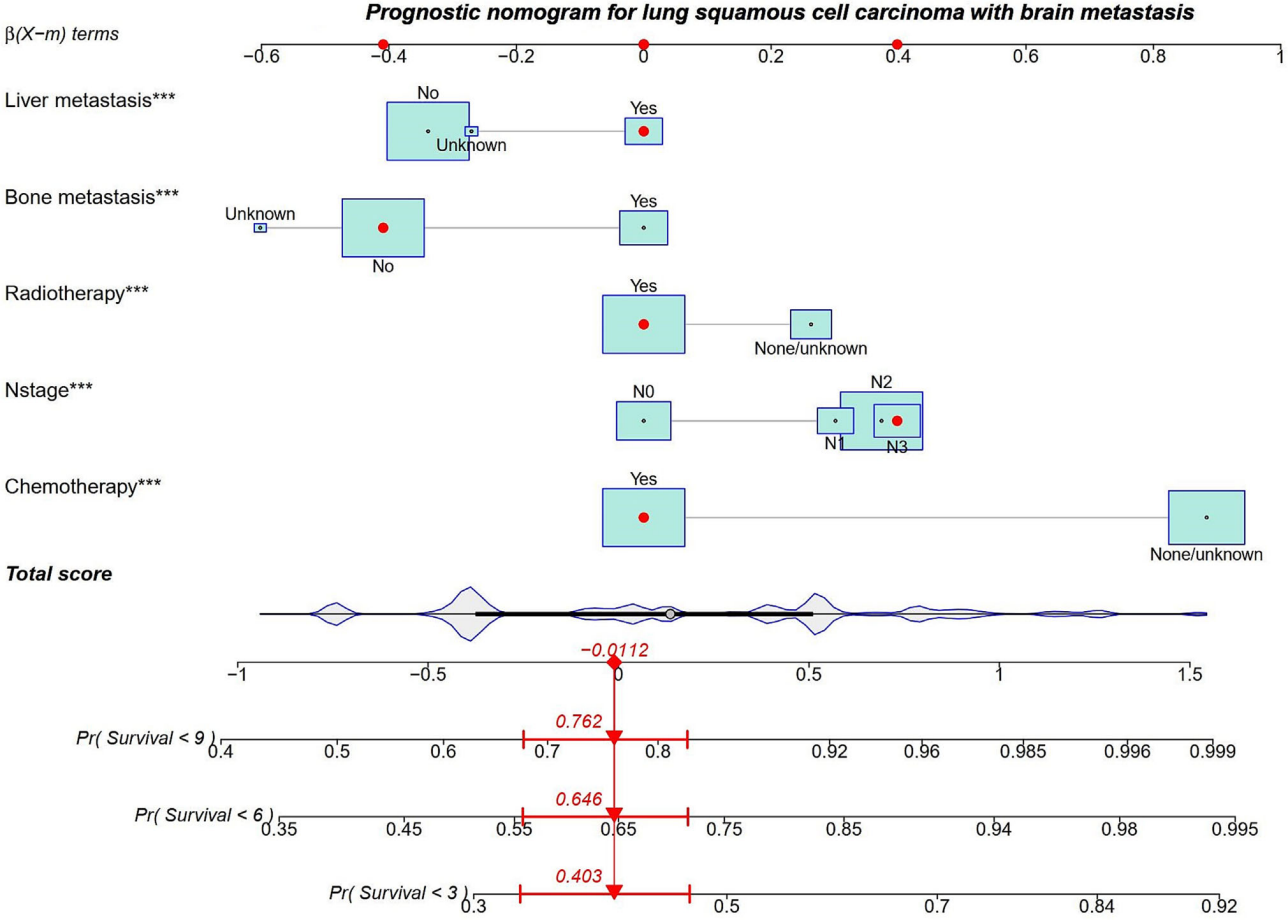
A novel nomogram for prediction of 3‐, 6‐, and 9‐month overall survival in patients who presented with brain metastases from lung squamous cell carcinoma.

According to the result of multivariate Cox proportional hazards analysis, parameters of N stage, bone metastasis, liver metastasis, radiotherapy, and chemotherapy were selected for nomogram development. In the nomogram, the variable values in the model will be cleared according to the status of the patient, so each variable will indicate a point value. The nomogram illustrated that chemotherapy had the most considerable contribution to prognosis, with a point score of nearly 1, followed by N stage and radiotherapy. The total risk score of an individual patient is calculated by adding the single points for each of the five items, and by summing the total score and finding where it falls on the survival scale, we can draw a vertical line downwards from this point and identify the 3‐, 6‐, and 9‐month survival probabilities of LUSC (Figure 2).

### 3.4 Model performance and validation

**FIGURE 3:**
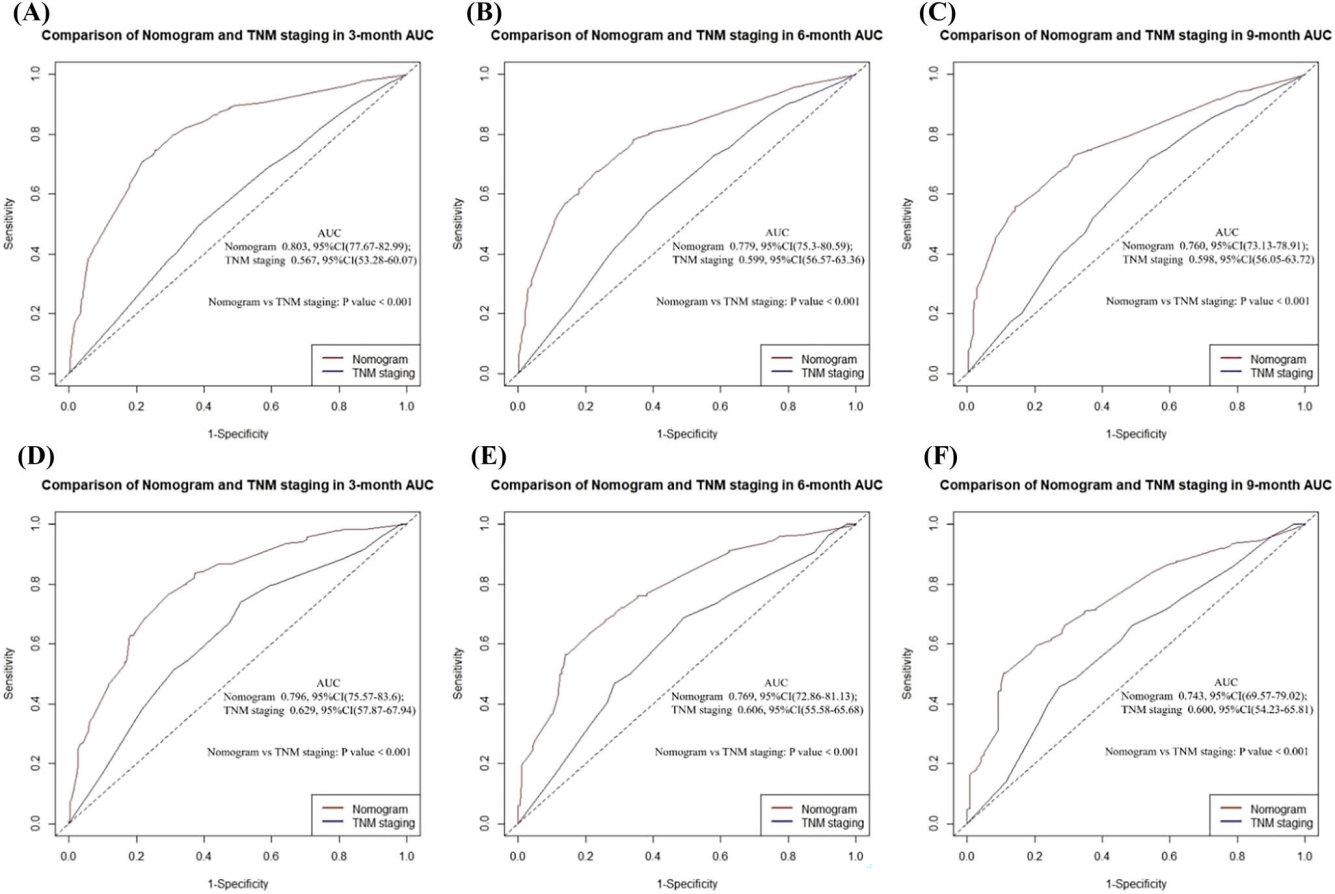
Comparison of nomogram and TNM staging for 3‐, 6‐, and 9‐month overall survival prediction in the population: The receiver operating characteristic curve (ROC) predicts (A) 3‐, (B) 6‐, and (C) 9‐month overall survival in the training cohort; ROC predicts (D) 3‐, (E) 6‐, and (F) 9‐month overall survival in the validation cohort.

In the training cohort, the AUCs for the developed nomogram were 0.803 (95% confidence interval [CI] 77.67–82.99), 0.779 (95% CI 75.3–80.59), and 0.760 (95% CI 73.13–78.91) for 3‐, 6‐, and 9‐month OS, respectively. Whereas in the validation cohort, the AUCs for the developed nomogram were 0.796 (95% CI 75.57–83.6), 0.769 (95% CI 72.86–81.13), and 0.743 (95% CI 69.57–79.02) for 3‐, 6‐, and 9‐month OS, respectively. Additionally, we compared AUCs between our nomogram and the AJCC‐TNM staging systems with the DeLong test to determine whether our model is predictive. As is shown in Figure 3, in the training cohort, AUCs predicting the nomogram's 3‐, 6‐, and 9‐month OS were significantly higher than the ones for the AJCC‐TNM staging system (*P* < 0.001). We obtained similar results in the validation cohort while comparing our nomogram with the AJCC‐TNM staging system in terms of predicting 3‐, 6‐, and 9‐month survival. In addition, to investigate the dynamic predictive power of our model in OS prediction, we plotted time‐dependent ROC curves based on the data from the two cohorts (Figure S1). The result highlighted the robust predictive power of our model compared to the TNM staging system. Together, these results demonstrate that our nomogram has significant prognostic value.

**FIGURE 4:**
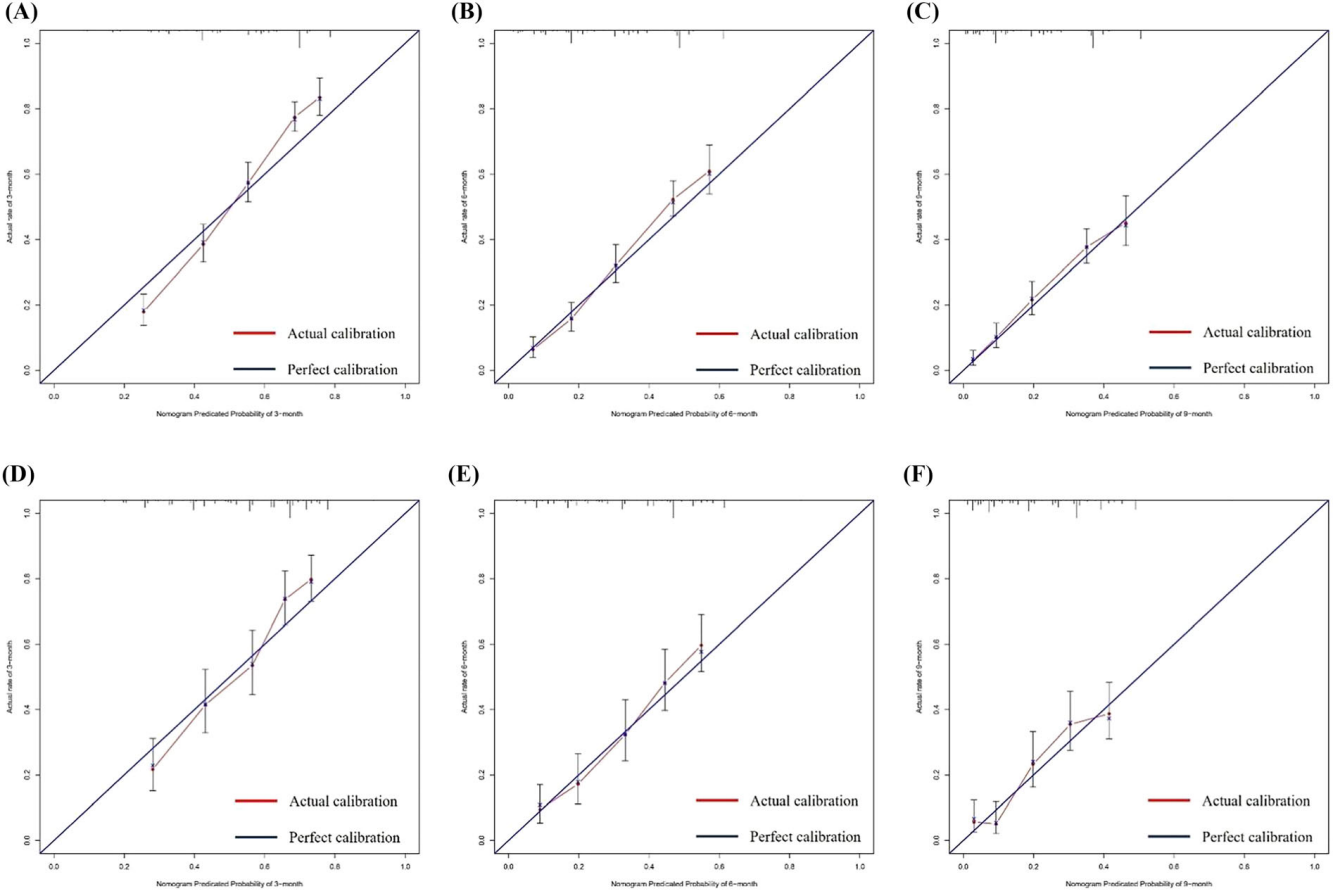
Calibration plots for nomogram‐predicted overall survival (*x*‐axis) and actual observed survival (*y*‐axis) in the population: calibration plots for (A) 3‐, (B) 6‐, and (C) 9‐month overall survival in the training cohort. Calibration plots for (D) 3‐, (E) 6‐, and (F) 9‐month overall survival in the validation cohort.

**FIGURE 5:**
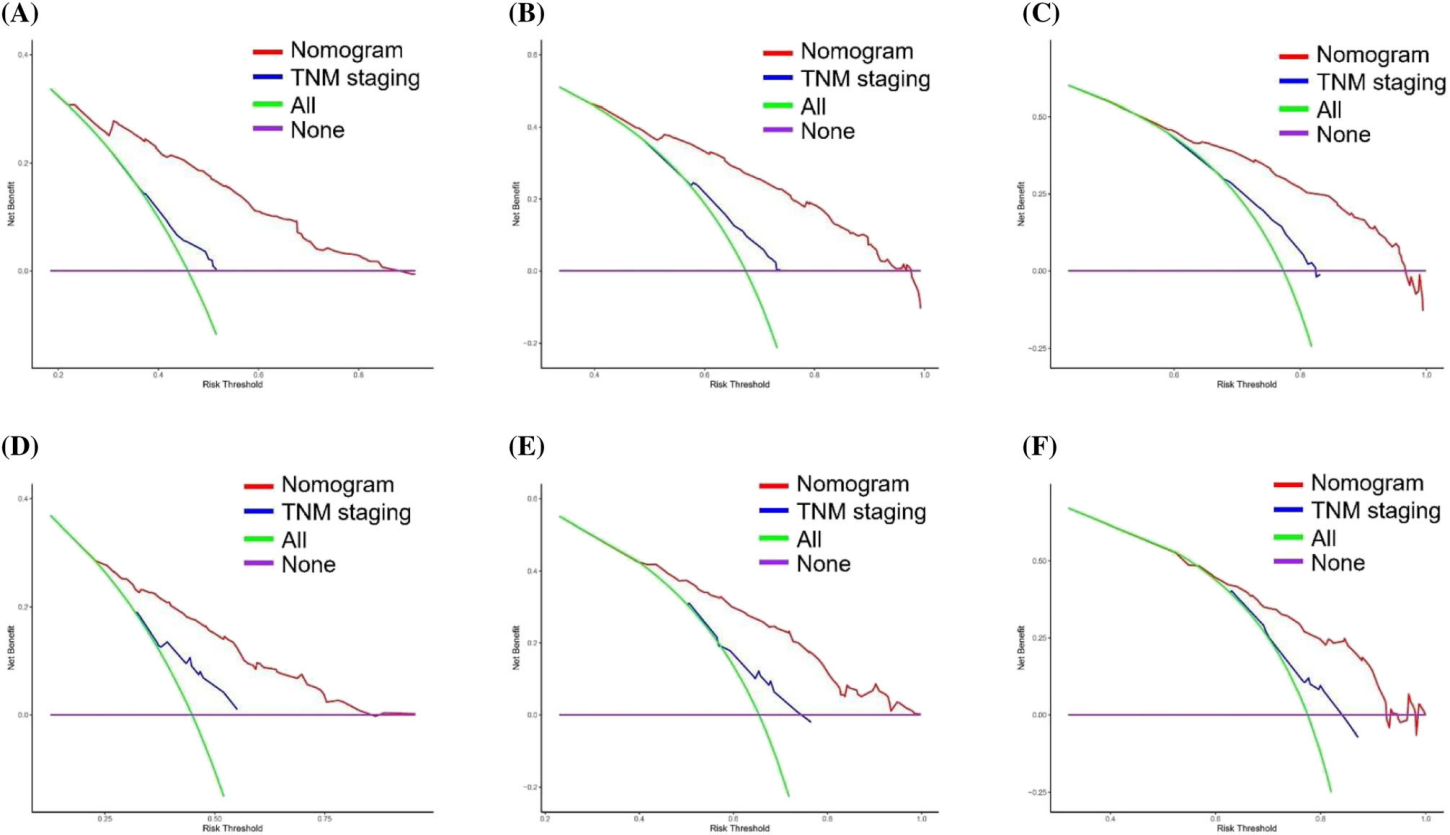
Decision curve analysis on the predictive model: decision curve analysis for (A) 3‐, (B) 6‐, and (C) 9‐month overall survival in the training cohort. Decision curve analysis for (D) 3‐, (E) 6‐, and (F) 9‐month overall survival in the validation cohort. The *x*‐axis represents the threshold probabilities, and the *y*‐axis represents the net benefit.

As can be found in Figure 4, according to calibration plots, the 3‐, 6‐, and 9‐month survival rates in training and validation cohorts were excellently consistent with nomogram predictions. Additionally, our nomogram showed a superior advantage when compared with the AJCC‐TNM staging system and could be of significant clinical utility (Figure 5).

### 3.5 Development of the risk classification system

**FIGURE 6:**
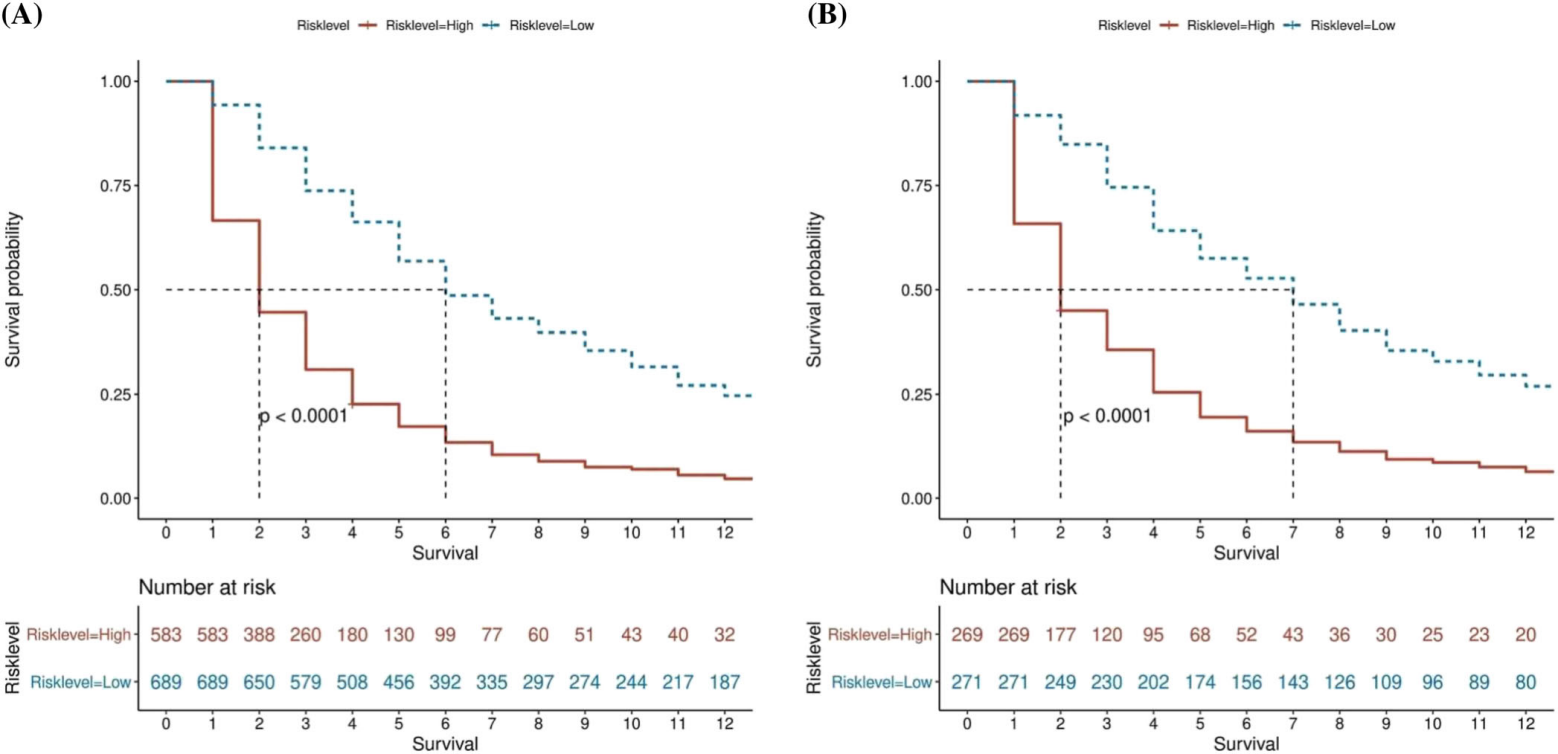
Kaplan–Meier (KM) curve analyses grouped by the risk classification system in the (A) training cohort and (B) validation cohort.

To provide a quantitative tool for predicting risk classification, a score predictive model derived from the nomogram was proposed in the training cohort. Specifically, using the optimum cutoff value (1.25 points) obtained from the training cohort, patients were assigned to high‐risk and low‐risk groups, and their survival was explored. According to the analysis of the KM curves, there was a significant difference in survival between the two groups. The log‐rank test found the difference between the two groups significant (*P* < 0.001). Using the same grouping method, similar results were observed in the validation cohort as well (Figure 6).

### 3.6 Nomogram webserver development

An online version of our nomogram based on a user‐friendly website was developed to support its application in clinical practice (Figure S2). The website allows researchers and physicians to easily calculate the corresponding predicted survival odds by plugging in specific clinical data (https://prognosticmodel-lusc.shinyapps.io/Nomogram-LUSC-BM/).

## 4 DISCUSSION

In the current study, by utilizing a large sample size of patient data from a population‐based database, we developed and validated a prognostic nomogram to provide a prediction of individual survival for patients who presented with BMs from LUSC. With our easy‐to‐use online calculator, investigators and physicians can calculate individualized survival probabilities based on clinicopathological parameters and treatment information. It is therefore possible that our study may facilitate clinical decision‐making and help future trials be designed and interpreted more effectively.

To date, staging classification of lung cancers is conducted by the AJCC‐TNM system, which is considered the gold standard for the decision in choosing the therapy of cancers or predicting clinical parameters for prognosis. Unfortunately, the system is relatively nondiscriminatory, and even at the same stage, some patients show worse biological behaviors.^12^ Given that BM remains a devastating complication and therapeutic challenge in LUSC and because of the insufficient predictive power of TNM staging in cancer prognosis, prognostic models with robust and high predictive ability are warranted for this population. In recent years, more and more clinical parameters have been revealed to be associated with the clinical prognosis of various types of cancer. Such parameters would enhance the ability to predict survival on an individual basis if they were integrated into rationally designed predictive models. As an alternative to traditional methods, the nomogram has emerged as a more straightforward and advanced method,^13^, ^14^, ^15^, ^16^, ^17^ owing to its advantage in flexibly using clinical parameters to predict the incidence of adverse events or survival rate through a scoring system rather than any complicated calculation formula.

Currently, there are several nomograms available for consolidating and prognosticating brain metastasized patients' mortality risk. For instance, in 2018, Agarwal et al. developed a nomogram to predict OS among NSCLC patients who underwent whole‐brain radiotherapy.^18^ The study was prospectively designed based on a single‐institutional sample size of 140 patients. Their model achieved a C‐statistic of 0.64 in the derivation sample, which seems to be unsatisfactory for predicted probabilities. The relatively small sample size might have weakened the robustness of the model as well. In the prognostic nomograms that were developed for NSCLC based on the SEER database,^19^, ^20^, ^21^ the predictive capacity of cancer survival in the models ranged from AUC 0.62 to 0.80. Unfortunately, despite being well established in some of the above studies, the models failed to provide a clear interpretation of OS prediction in a specific type of NSCLC patient. In such cases, the determining factors associated with OS were suitable for the overall NSCLC rather than a single type of lung cancer. Given that lung cancer is a heterogeneous group of carcinomas with different biological behaviors and prognoses, confounding biases are likely to be inevitable when these different subtypes of lung cancer are taken together in time‐ and event‐based Cox regression analysis. The sources of these confounding biases were probably generated by the various determinants of OS among specific subtypes of lung cancer, such as tumor stage, degree of tumor differentiation, and sensitive treatments. In light of our nomogram's specific design for the brain metastasized LUSC population, such a model may provide more accurate survival estimates. Moreover, the vast geographical coverage of the database ensures the generalizability of our model for this population.

In regard to the prognosis of brain metastasized patients from LUSC based on clinical parameters, variables screened by our nomogram demonstrated that the most important independent prognostic factor was chemotherapy, followed by the N stage and radiotherapy. Moreover, distant metastases to bone and liver were also found to be associated with a poor prognosis. These findings are consistent with some milestone studies and clinical guidelines. However, it is interesting to note that age is not an independent prognostic factor for this population. This can be explained as LUSC usually has an extremely poor prognosis when encountering BMs. This notion is in line with our study, in which only a median follow‐up of 4 months was observed in this population. Because of the lack of emerging promising treatment approaches for lung cancer reported in the SEER database (e.g., target therapy and immune checkpoint inhibitors [ICIs]), the study failed to explore their role in cancer prognosis, therefore, failing to build a prognostic model by integrating these parameters. However, targeted therapy may not be sufficient given that LUSC has a low frequency of the targeted effective mutation. On the other hand, traditional anti‐cancer treatment such as chemoradiotherapy remains a cornerstone element for this population, as the efficacy of ICIs may differ greatly across patients, and the high price, along with the limited availability of ICIs, severely impedes their application in clinical practice.^22^

Referring to the model's performance, to ensure the reliability of our model, we validated and calibrated it to prevent overfitting and to verify that it is generalizable across the population. Based on the calibration curves, we can conclude that the model is valid, as the actual and model‐predicted survival probabilities were in excellent agreement. Further justifying the clinical utility of our model, we evaluated DCA curves to determine potential clinical effects. Compared with the AJCC‐TNM staging, our model outperformed in achieving higher net benefits, meaning that it had better clinical implementation significance. In addition, medical professionals are able to identify high‐risk patients on the basis of the risk classification system who may require additional treatment or intensive monitoring. Despite this, direct use of the scoring system may not be appropriate, as multiple complex factors (such as personal and financial) influence a doctor's treatment decisions.

Despite the successful establishment of a prognostic model with good predictive power among patients who presented with BMs from LUSC, this study may be subject to multiple limitations, such as study design, data collection, model validation, and interpretation. Firstly, we were unable to avoid selection bias because of the retrospective nature of our study. Secondly, despite being a large database, SEER is limited by the information it stores. For instance, a description of the specific type of surgery, radiation dose, and choice of chemotherapy drugs involved in systemic treatment. Additional information about distant metastases and comorbidities is also not available in the database. This current nomogram also needs to be updated with additional clinical data in light of the development of new treatments for patients with LUSC. Furthermore, we did not compare the 8th AJCC‐TNM staging to our model because there were insufficient eligible cases available in the database. Finally, although bootstrap resampling was applied to avoid overfitting, this model should still be externally validated.

## 5 CONCLUSIONS

This nomogram provides accurate estimates of survival probability in patients who presented with BMs from LUSC. Physicians can use it as a tool to identify patients with a high survival risk who may need treatment and intensive follow‐up to improve their prognosis.

## AUTHOR CONTRIBUTIONS

*Study concepts*: Min Liang and Shivank Singh. *Study design*: Min Liang and Mafeng Chen. *Data acquisition*: Min Liang and Caijian Zhou. *Data analysis and interpretation*: Min Liang and Shivank Singh. *Manuscript preparation*: Min Liang and Shantanu Singh. All named authors meet the International Committee of Medical Journal Editors (ICMJE) criteria for authorship for this article, take responsibility for the integrity of the work as a whole, and have given their approval for this version to be published.

## CONFLICT OF INTEREST STATEMENT

All authors declare that they have no competing interests.

## COMPLIANCE WITH ETHICS GUIDELINES

This article was built on open‐access databases and did not contain any new research involving human participants or animals. The ethics committee approved the protocol for Maoming People's Hospital study. All authors have signed the SEER Research Data Agreement to protect the privacy of patients, which is consistent with ethical principles.

## Supporting information


**Figure S1.** Dynamic ROC curves for predicting overall survival in lung squamous cell carcinoma patients with brain metastases. Predicting overall survival in the training cohort(A) and validation cohort(B). The follow‐up period was measured in months.
**Figure S2.** Online web server interface for the prognostic nomogram.

## Data Availability

The datasets generated and analyzed during the current study are available in the SEER repository (https://seer.cancer.gov/data/).
