# Supplementary material for: A visualized dynamic prediction model for overall survival in patients diagnosed with brain metastases from lung squamous cell carcinoma
Source: Clin Respir J. 2023 Apr 29;17(6):556–67. doi: 10.1111/crj.13625 (PMC10265177; doi:10.1111/crj.13625)
Supplement: Supplementary file 1 — Figure S1. Dynamic ROC curves for predicting overall survival in lung squamous cell carcinoma patients with brain metastases. Predicting overall survival in the training cohort(A) and validation cohort(B). The follow‐up period was measured in months. Figure S2. Online web server interface for the prognostic nomogram. [file CRJ-17-556-s001.docx]

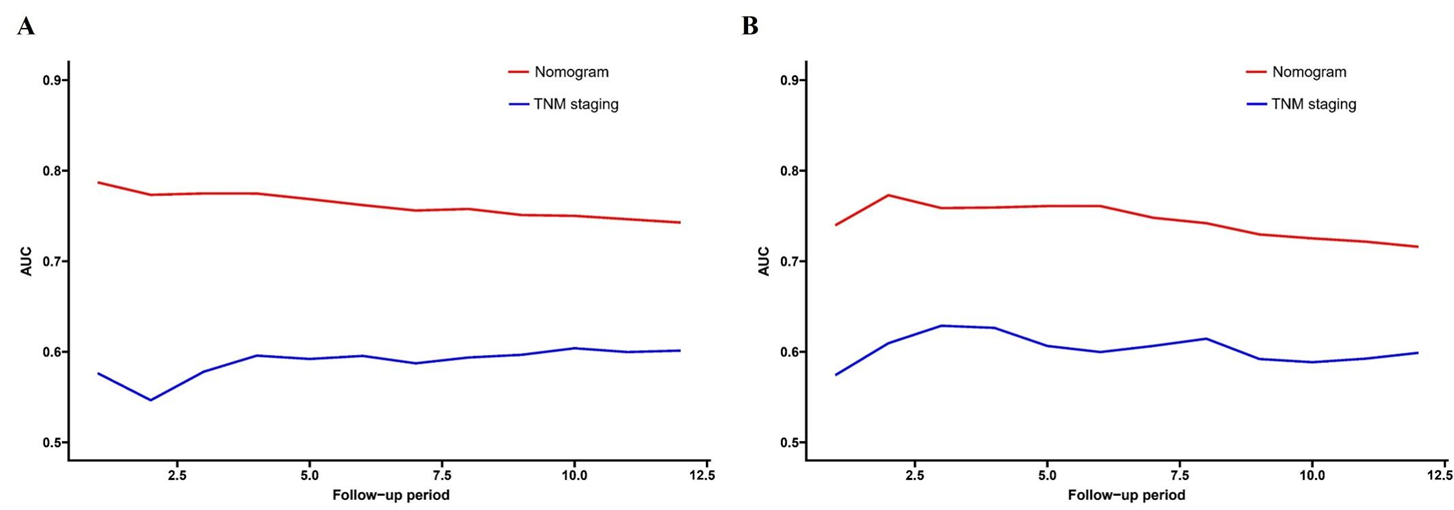


**Supplementary Figure1.** Dynamic ROC curves for predicting overall survival in lung squamous cell carcinoma patients with brain metastases. Predicting overall survival in the training cohort(A) and validation cohort(B). The follow-up period was measured in months.


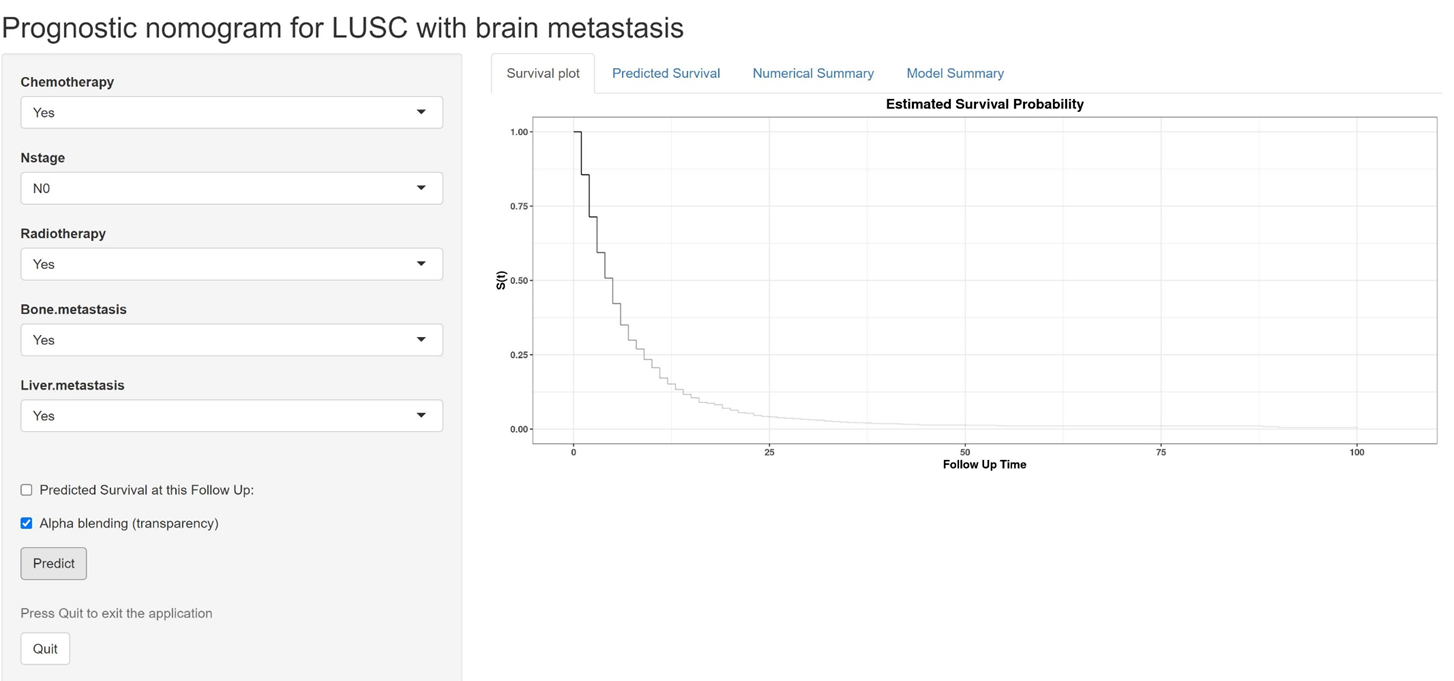


**Supplementary Figure2.** Online web server interface for the prognostic nomogram.
